# Multimodal assessment of painful peripheral neuropathy induced by chronic oxaliplatin-based chemotherapy in mice

**DOI:** 10.1186/1744-8069-7-29

**Published:** 2011-04-26

**Authors:** Cynthia L Renn, Valentina A Carozzi, Peter Rhee, Danisha Gallop, Susan G Dorsey, Guido Cavaletti

**Affiliations:** 1 School of Nursing, Center for Pain Studies, University of Maryland, Baltimore, MD, USA; 2 Department of Neuroscience and Biomedical Technologies Department, University of Milan Bicocca, Monza (MB), Italy

**Keywords:** Oxaliplatin, peripheral neuropathy, cold hyperalgesia, mechanical allodynia, dorsal root ganglia, spinal dorsal horn, electrophysiology

## Abstract

**Background:**

A major clinical issue affecting 10-40% of cancer patients treated with oxaliplatin is severe peripheral neuropathy with symptoms including cold sensitivity and neuropathic pain. Rat models have been used to describe the pathological features of oxaliplatin-induced peripheral neuropathy; however, they are inadequate for parallel studies of oxaliplatin's antineoplastic activity and neurotoxicity because most cancer models are developed in mice. Thus, we characterized the effects of chronic, bi-weekly administration of oxaliplatin in BALB/c mice. We first studied oxaliplatin's effects on the peripheral nervous system by measuring caudal and digital nerve conduction velocities (NCV) followed by ultrastructural and morphometric analyses of dorsal root ganglia (DRG) and sciatic nerves. To further characterize the model, we examined nocifensive behavior and central nervous system excitability by *in vivo *electrophysiological recording of spinal dorsal horn (SDH) wide dynamic range neurons in oxaliplatin-treated mice

**Results:**

We found significantly decreased NCV and action potential amplitude after oxaliplatin treatment along with neuronal atrophy and multinucleolated DRG neurons that have eccentric nucleoli. Oxaliplatin also induced significant mechanical allodynia and cold hyperalgesia, starting from the first week of treatment, and a significant increase in the activity of wide dynamic range neurons in the SDH.

**Conclusions:**

Our findings demonstrate that chronic treatment with oxaliplatin produces neurotoxic changes in BALB/c mice, confirming that this model is a suitable tool to conduct further mechanistic studies of oxaliplatin-related antineoplastic activity, peripheral neurotoxicity and pain. Further, this model can be used for the preclinical discovery of new neuroprotective and analgesic compounds.

## Background

Oxaliplatin is an effective platinum-based drug used as first line chemotherapy for metastatic colorectal cancer [1]. Moreover it has been used to treat some cisplatin-resistant cancers, including those of the stomach [2], pancreas [3], ovary [4], breast and lung [5]. Oxaliplatin induces DNA crosslinks that cause apoptotic death of dividing cells [6] and reduced tumor growth. Unfortunately, the platinum derivative drugs have a molecular affinity for the peripheral nervous system [7,8], leading to severe peripheral neurotoxicity that affects most cancer patients treated with oxaliplatin-based chemotherapy. Oxaliplatin-induced peripheral neuropathy is clinically characterized by two different types of neurological symptoms [9]. One type, occurring in 90% of patients, is an acute, transient syndrome characterized by cramps, paresthesias and dysesthesias that are triggered or enhanced by exposure to cold. The second type is a chronic [9] and more severe syndrome that is characterized by the loss of sensory perception and frequently associated with painful sensations that generally occur after repeated drug administration. The mechanisms underlying the development of oxaliplatin-induced neurotoxicity remain unclear.

Several studies have examined the neurophysiological, behavioral and pathological characteristics of oxaliplatin-induced peripheral neurotoxicity using rat models [10] and most of the oxaliplatin-induced pain studies have been done after a single injection of the drug. While rats developed significant cold and mechanical allodynia following a single dose of oxaliplatin, these models are not representative of the chronic neurotoxicity experienced in clinical practice [11,12]. Cavaletti et al. [7] demonstrated that chronic oxaliplatin treatment in rats induced atrophy of dorsal root ganglia (DRG) neurons and decreased peripheral sensory nerve conduction velocities (NCV). Moreover, chronic oxaliplatin treatment induced cold and heat hypersensitivity along with mechanical allodynia that persisted for 3 weeks after drug treatment ended [13]. The use of rat models to study oxaliplatin-induced neurotoxicity has been very informative. However, since it is difficult to implant tumors in rats, most studies of the anticancer properties of oxaliplatin have used mice. Thus, rat models have limited efficacy for investigations of peripheral neurotoxicity in the same experimental paradigms used to evaluate the anticancer activity of oxaliplatin.

Recently, several mouse models of oxaliplatin-induced pain have been developed using an acute, single dose [14,15] or chronic, repeated doses of oxaliplatin [15]. While these studies demonstrated the development of mechanical and cold allodynia after oxaliplatin treatment [14,15], the characterization of peripheral neurotoxicity was limited. To address these limitations we have performed this study in BALB/c mice treated with a schedule of oxaliplatin able to induce the onset of a painful neuropathy with the aim to achieve a more complete characterization of the peripheral and central nervous system events induced by the chronic treatment.

## Results

### 1. General Appearance and Body Weight Change

**Figure 1:**
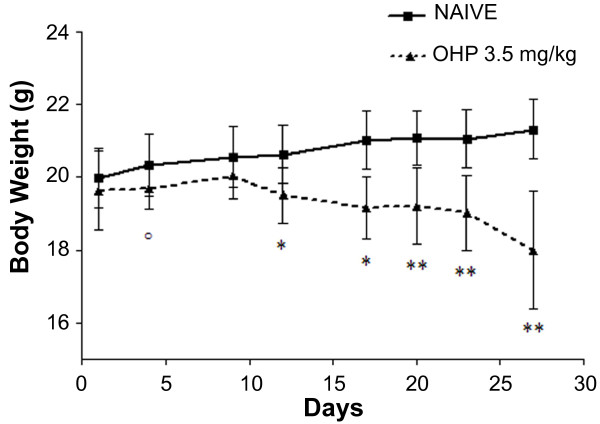
**Body weight decreases after oxaliplatin (OHP) treatment**. Mice treated with oxaliplatin3.5 mg/kg/iv twice weekly (n = 8) lost a significant amount of body weight compared to naïve mice (n = 8) °p < 0.05, * p < 0.01, ** p < 0.0001 vs. naïve, ANOVA with repeated measures.

To generate the model of oxaliplatin-induced painful peripheral neuropathy used in this study, the mice were given tail vein injections of oxaliplatin (3.5 mg/kg) twice weekly (separated by either 3 or 4 days) for four weeks. The control group was naïve mice that did not receive drug or vehicle injections. The duration of this study was 30 days, during which the mice were continuously allodynic after receiving oxaliplatin. The oxaliplatin was generally well-tolerated by the mice. They continued to groom, make nests, explore their surroundings and climb on their wire cage tops during the course of drug treatment, though approximately 20% showed signs of mild kyphosis and piloerection. The mice were weighed on drug administration days and, over the course of the study, the oxaliplatin-treated mice had a significant decrease in body weight compared to the naïve mice (Figure 1; day 4, p < 0.05; days 12 and 17, p < 0.01; days 20, 23, 27, p < 0.0001), reaching 15% by the completion of the study. No mice were euthanized prematurely during the course of the study.

### 2. Nocifensive Response After Oxaliplatin

It is well established that oxaliplatin treatment causes peripheral neuropathy in humans and neuropathic-like changes in rats. To determine whether our mouse model of chronic oxaliplatin treatment-induced neurotoxicity also exhibited signs of pain, the mice were tested for the development of nocifensive responses to mechanical and thermal stimuli. The mice were randomly assigned to an experimental group and tested for their baseline nocifensive responses. After the onset of oxaliplatin treatment, the mice were tested after the second dose of drug each week for four weeks.

**Figure 2:**
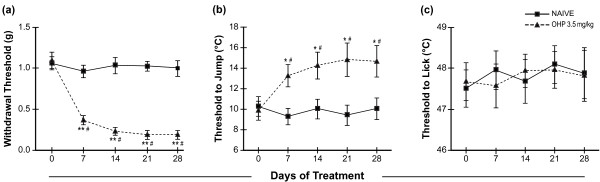
**Oxaliplatin (OHP) induces mechanical and cold allodynia but not heat hyperalgesia**. (a) Mice treated with oxaliplatin 3.5 mg/kg/iv twice weekly (n = 8) had a significant decrease in mechanical threshold from baseline and compared to naïve mice (n = 8) that started after the first week of treatment and persisted for at least four weeks. **p < 0.001 vs. baseline, Friedman Test; #p < 0.001 vs. naïve, Mann Whitney U Test. (b) Mice treated with oxaliplatin 3.5 mg/kg/iv twice weekly (n = 8) had a significant increase in cold threshold from baseline and compared to naïve mice (n = 8) that started after the first week of treatment and persisted for at least four weeks. *p < 0.01 vs. baseline, ANOVA with Repeated Measures; #p < 0.05 vs. naïve, Student's T Test. (c) Mice treated with oxaliplatin 3.5 mg/kg/iv twice weekly (n = 8) had no change heat threshold from baseline and were not different from naïve mice throughout the duration of the experiment. P > 0.05 vs. baseline, ANOVA with Repeated Measures; p > 0.05 vs. naïve, Student's T Test.

Mechanical allodynia was defined as a decrease in paw withdrawal threshold (g) from baseline. The oxaliplatin-treated mice had a significant decrease in mechanical threshold after the first week of oxaliplatin treatment compared to baseline that persisted for at least four weeks (Figure 2a; Chi Sq. 17.36, df 4, p < 0.001), while the naïve mice did not (Chi Sq. 0.49, df 4, p > 0.05). Further, the oxaliplatin-treated mice had a significantly lower mechanical threshold than the naïve mice after the first week of oxaliplatin treatment that persisted for at least four weeks (p < 0.001).

Cold allodynia was defined as an increase in the threshold temperature (°C) that elicited a jumping response compared to baseline. The oxaliplatin-treated mice had a significant increase in threshold temperature after the first week of oxaliplatin treatment compared to baseline that persisted for at least four weeks (Figure 2b; F = 4.65, df 4, p < 0.01), while the naïve mice did not (F = 0.25, df 4, p = 0.91). Further, the oxaliplatin-treated mice had a significantly higher threshold temperature than the naïve mice after the first week of oxaliplatin treatment that persisted for at least four weeks (p < 0.05). By contrast, there was no evidence of heat hyperalgesia (Figure 2c), which was defined as an increase in the threshold temperature (°C) that elicited a hind paw licking response. There was no change in heat threshold from baseline for either the oxaliplatin-treated (F = 0.11, df 4, p = 0.97) of naïve (F = 0.19, df 4, p = 0.94) throughout the four weeks. Further, there was no difference in heat threshold between groups (p > 0.05).

### 3. NCV, Neuropathological Analysis of DRG and Sciatic Nerve

The first series of experiments demonstrate that mice chronically treated with oxaliplatin are not significantly debilitated by the drug treatment; however, chronic oxaliplatin treatment does induce sensitivity to mechanical and cold stimuli. Thus, this is a valid model to study chronic oxaliplatin treatment-induced allodynia. Next we used morphometry and NCV measurements to determine whether this mouse model of chronic oxaliplatin treatment exhibited neurotoxic changes in the structure and function of peripheral neurons.

#### 3.1. NCV

**Figure 3:**
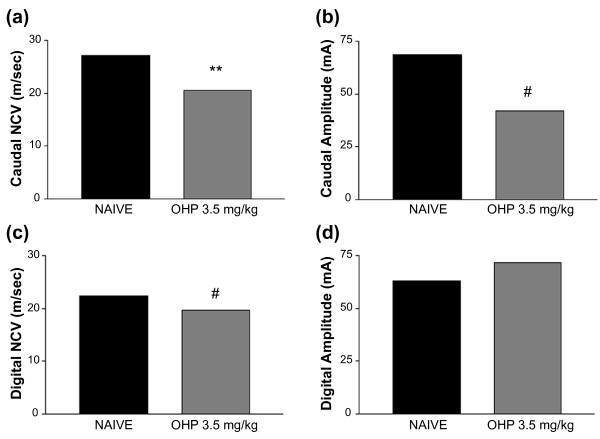
**Oxaliplatin (OHP) decreases NCV in caudal and digital nerves**. (a) Mice treated with oxaliplatin 3.5 mg/kg/iv twice weekly for four weeks (gray bars; n = 8) had a significant decrease in caudal NCV compared to naïve mice (black bars; n = 8). (b) The oxaliplatin-treated mice had a significant decrease in caudal nerve action potential amplitude compared to naïve mice. (c) The oxaliplatin-treated mice had a significant decrease in digital NCV compared to naïve mice. (d) The oxaliplatin-treated mice had no difference in the amplitude of the digital nerve action potential compared to naïve mice. **p < 0.0001 vs. naive, #p < 0.001 vs. naive, Student's T Test.

After establishing that this mouse model exhibits a pain phenotype, we next wanted to determine whether alterations in peripheral nerve function could be contributing to the development of oxaliplatin-induced allodynia. To assess the functional status of peripheral neurons after chronic oxaliplatin treatment, the mice were randomly assigned to experimental groups. NCV and action potential amplitude were measured in the caudal and digital nerves 4 days after the final oxaliplatin dose in week four (Figure 3). Chronic oxaliplatin treatment induced a significant decrease in the caudal NCV (Figure 3a; p < 0.0001) with a concomitant significant decrease in action potential amplitude (Figure 3b; p < 0.01) compared to the naïve group. After oxaliplatin, the digital NCV also significantly decreased (Figure 3c; p < 0.001), though the action potential amplitude was not different (Figure 3d), compared to the naïve group.

#### 3.2. Morphological Analysis of DRG and Sciatic Nerve

**Figure 4:**
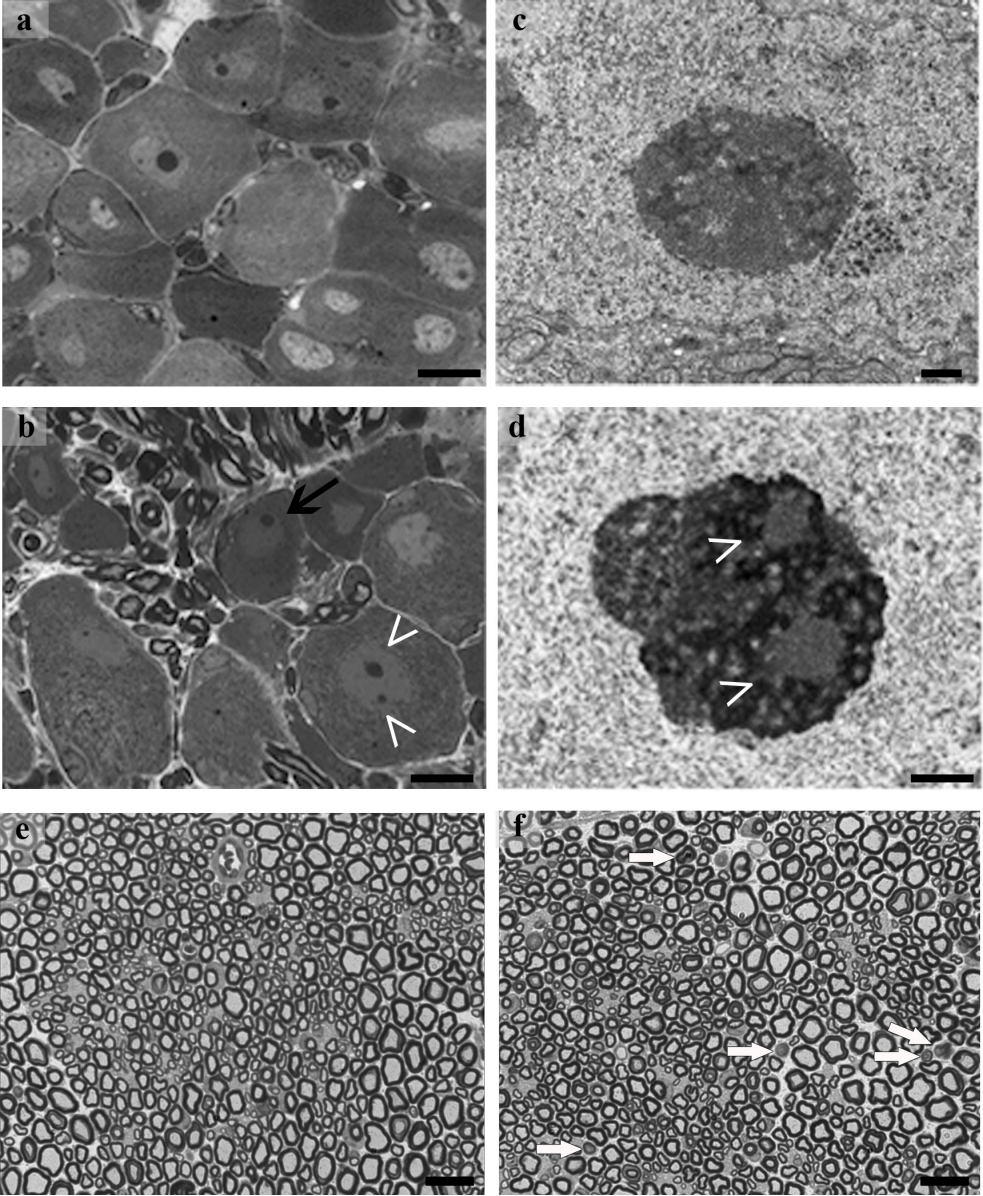
**Oxaliplatin induces nucleolar segregation and axonopathy**. (a, b) Light microscopy revealed that DRG neurons from mice treated with oxaliplatin 3.5 mg/kg/iv twice weekly for four weeks (b; n = 3) had segregated nucleoli (arrow heads) that were eccentric (black arrow) compared to DRGs from naïve mice (a; n = 3). (c, d) Electron microscopy also showed segregated nucleoli in DRG neurons from oxaliplatin-treated mice (d; n = 3) but not naïve mice (c; n = 3). Light microscope examination demonstrated that myelinated fibers in the sciatic nerve of mice treated with oxaliplatin 3.5 mg/kg/iv twice weekly for four weeks (f; n = 3) had evidence of changes indicative of axonopathy (white arrows) that were not found in sciatic nerve from naïve mice (e; n = 3).

Next, we wanted to determine whether the altered function of peripheral neurons after oxaliplatin was accompanied by structural changes in the DRG cell bodies and axons of the sciatic nerve. For this purpose, thin sections through the L4-L5 DRGs and sciatic nerve from naïve and oxaliplatin-treated mice were examined at the light and electron microscope levels two days after the final dose of drug in week four (Figure 4). Light microscopy revealed that DRG neurons from oxaliplatin-treated mice had a high incidence of multinucleolated cell bodies (Figure 4b arrowheads) with eccentric nucleoli (Figure 4b black arrow) compared to naïve DRG neurons (Figure 4a). In both experimental groups, the cytoplasm of neurons and satellite cells appears normal. The small circular structures with black borders that are evident between the cell bodies in Figure 4b are radicular fibers crossing the DRG. These findings were verified by electron microscopy, which showed nucleolar segregation in DRG neurons from oxaliplatin-treated (Figure 4d arrowheads) but not naïve mice (Figure 4c). Examination of sciatic nerves by light microscope showed that myelinated fibers in the sciatic nerve of oxaliplatin-treated mice (Figure 4f, white arrows) had mild changes indicative of axonopathy, which were not evident in sciatic nerve from naïve mice (Figure 4e). There were no changes evident in the unmyelinated fibers from either group.

**Figure 5:**
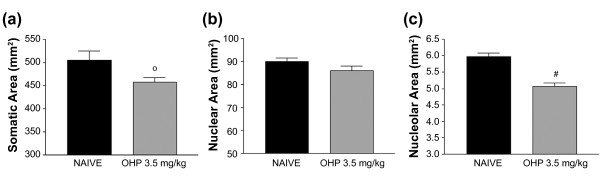
**Oxaliplatin (OHP) treatment causes decreased the area of DRG cell body and nucleolus**. Morphometric analysis revealed that DRG neurons from mice treated with oxaliplatin 3.5 mg/kg/iv twice weekly for four weeks (n = 3) had a significant decrease in the area (mm^2^) of the cell bodies (a) and nucleoli (c) compared to DRG neurons from naïve mice (n = 3). (b) There was no difference in the areas of the nuclei from oxaliplatin-treated and naïve mice. °p < 0.05 vs naïve, #p < 0.001 vs naïve, Student's T Test.

Our previous work in rat models demonstrated that platinum-derived compounds induce DRG neuron cell body shrinkage [7,16,17]. Since finding that oxaliplatin induced changes to the nucleoli of DRG neurons in our mouse model, we performed a morphometric analysis to examine the cell bodies of DRG neurons from oxaliplatin-treated (3.5 mg/kg/iv twice weekly for four weeks) and naïve control mice for evidence of cell body shrinkage similar to that seen in rats. The morphometric analysis revealed that DRG neurons from oxaliplatin-treated mice (gray bars) had a significant decrease in the area (mm^2^) of their cell bodies (Figure 5a; p < 0.05) and nucleoli (Figure 5c; p < 0.001), but not nuclei, compared to DRG neurons from naïve mice (black bars).

### 4. Electrophysiological Analysis of Wide Dynamic Range Neurons in the Spinal Dorsal Horn

**Figure 6:**
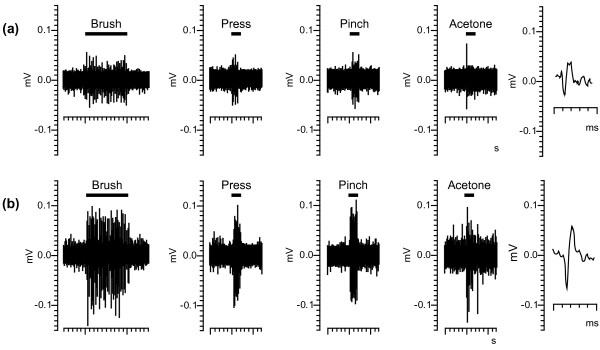
**Representative raw data trace of wide dynamic range neurons**. Example raw data used to construct a histogram of the stimulus-response to brush (10 second stimulus), pressure (2 second stimulus) and pinch (2 second stimulus) of an individual wide dynamic range neuron in a naïve mouse (a) or 2 days after the final dose of oxaliplatin 3.5 mg/kg/iv in the fourth week (b). The waveforms to the right of each trace show a representative spike that was analyzed.

**Figure 7:**
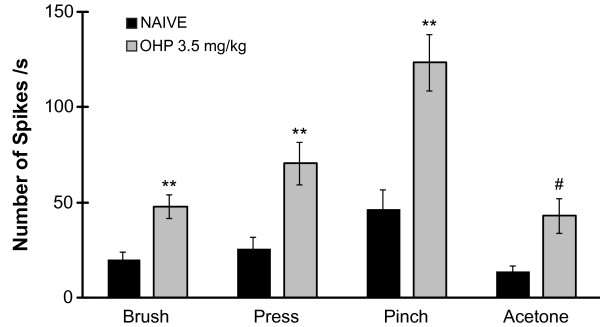
**Oxaliplatin (OHP) increases activity of wide dynamic range neurons in the spinal dorsal horn**. Two days after the final injection of oxaliplatin 3.5 mg/kg/iv, the peak number of spikes per second were higher in the oxaliplatin-injected (gray bars) mice compared with naïve mice (black bars). **p < 0.001 and #p < 0.01 vs. naive, Student's T Test.

After determining that chronic oxaliplatin treatment resulted in altered nocifensive behavior, peripheral nerve function and structural changes in DRG neuron cell bodies and sciatic nerve, our next question was to determine if there was a concomitant change in the activity of wide dynamic range neurons in the spinal dorsal horn. Two days after the mice received their final dose of oxaliplatin (3.5 mg/kg/iv twice weekly) in the fourth week, the activity of 43 deep dorsal horn neurons (20 neurons from 6 naive and 23 neurons from 6 oxaliplatin-treated mice; Table 1) was recorded and analyzed. Neurons were classified as wide dynamic range based on their response to innocuous and noxious mechanical stimuli applied to the plantar surface of the hind paw ipsilateral to the recording site (Figure 6). During stimulation, the number of spikes per second (Figure 7; Table 1) was significantly higher in the oxaliplatin-treated mice during the innocuous brush (64.93 ± 6.27 spikes/s), moderate pressure (70.59 ± 11.17 spikes/s), noxious pinch (123.38 ± 14.77 spikes/s) and acetone (43.09 ± 9.16 spikes/s) stimuli compared to the naive mice (24.47 ± 4.62, 25.78 ± 5.92, 46.56 ± 9.97, 13.92 ± 3.01 spikes/s respectively; p < 0.001 for brush, press, pinch; p < 0.01 for acetone).

**Table 1:** Oxaliplatin increases activity of wide dynamic range neurons in the spinal dorsal horn.

Treatment	Mice	Neurons	Brush		Press		Pinch		Acetone	
			Spikes	(SEM)	Spikes	(SEM)	Spikes	(SEM)	Spikes	(SEM)
**Naive**	n = 6	n = 20	24.47	(4.62)	25.78	(5.92)	46.56	(9.97)	13.92	(3.01)
**Oxaliplatin**	n = 6	n = 23	64.93	(6.27)**	70.59	(11.17)**	123.38	(14.77)**	43.09	(9.16) #

The peak number of spikes per second were higher in the oxaliplatin-injected mice compared with naïve mice. **p < 0.001 and #p < 0.01 vs. naive, Student's T Test.

## Discussion

Recent advances in cancer chemotherapy have significantly increased the survival rate and time of cancer patients, although the use of these drugs is also associated with an increase in morbidity related to the development of painful peripheral neuropathy [18-20]. Oxaliplatin, a third generation platinum derivative, is one of the most effective chemotherapeutic drugs used to treat advanced colorectal cancer [21-25]. Unfortunately, oxaliplatin therapy is associated with significant side effects such as neurotoxicity, which is one of the most prevalent and dose-limiting effects [26-28], occurs in greater than 65% of patients and includes mechanical allodynia and hypersensitivity to cold [29-31]. Oxaliplatin-induced peripheral neuropathy (OIPN) occurs in two forms, acute and chronic. The onset of acute OIPN occurs either during or within 1-2 days after drug infusion, resolves between cycles and recurs with subsequent infusions [25,27]. Chronic OIPN develops as a cumulative, dose-dependent effect [22,27,32,33] in patients that receive more than 540 mg/m^2^, regardless of the dosing regimen [23]. The symptoms of chronic OIPN can be debilitating, persistent and respond poorly to currently available therapeutics. Thus, it is critical that we gain a better understanding of the mechanisms underlying the development and persistence of chronic OIPN, which could lead to the development of new treatment modalities to improve symptom management.

The majority of studies examining the neurotoxic effects of oxaliplatin treatment have been done after treatment in the rat [7,11,13,34], while the majority of studies examining the antineoplastic efficacy of oxaliplatin have been done in mouse. Recently, though, several studies have been done to examine oxaliplatin-induced neurotoxicity in the mouse [14,15]. However, the mouse studies have not closely examined the neurotoxic effects of chronic oxaliplatin treatment on the status of peripheral nerves and spinal dorsal horn neuronal activity. In this study, we used a mouse model of oxaliplatin-induced neurotoxicity, based on our preliminary studies in the rat [7,35], to investigate the effects of chronic oxaliplatin treatment on nocifensive behavior, peripheral nerve function and wide dynamic range neuron activity in the spinal dorsal horn. The dose of oxaliplatin and the administration schedule were chosen based on previous work in the rat [7] and preliminary pilot studies in the mouse. The mice received 3.5 mg/kg/iv twice weekly for four weeks, which is equivalent to 130 mg/m^2 ^per administration and a cumulative dose of 1080 mg/m^2 ^after 8 cycles. This dose of oxaliplatin and number of cycles has been found to cause neuropathy symptoms in patients [23,33,36]. The oxaliplatin treatment did not affect the general health and functioning of the mice, as assessed by appearance and activity, though the mice did lose weight during the course of the study. Although the weight loss was significant, it was tolerable and consistent with similar rat models [7]. Moreover, the association between the extent of weight loss and peripheral neuropathy has been ruled out, at least in the rat [37]. However, oxaliplatin treatment did produce significant mechanical and cold allodynia that persisted for the four week duration of the study, similar to symptoms reported by patients [1,9] and to what has been found in previous studies in the rat [12,13,11,38,39] and mouse [14,15,40]. Thus, this mouse model of oxaliplatin-induced neurotoxicity is representative of the human clinical condition and can be a useful tool to study the mechanisms that underlie the development and persistence of chronic OIPN.

Oxaliplatin-induced neuropathy has been associated with damage to the peripheral sensory neurons [7,41], which leads to alterations in peripheral nerve function [31,42]. In this study, we found that chronic oxaliplatin treatment induced a decrease in conduction velocities in the caudal and digital nerves that was associated with a concomitant decrease in caudal action potential amplitude. These results are similar to the findings of studies that were done in the rat [7,43]. Nucleolar morphology changes were also seen in this study that are similar to those shown previously [44]. The mechanisms of these decreases remain unclear and require further study; however, as suggested by Jamieson and colleagues, one possibility is that oxaliplatin induces a decrease in phosphorylated neurofilaments in DRG neurons with a concomitant alterations in sensory axons [45]. Following the finding that oxaliplatin induced a decrease in NCV and action potential amplitude; we examined the morphology of DRG neurons and sciatic nerve at the light and electron microscope levels. Our findings that the diameter of DRG cell bodies is reduced after chronic oxaliplatin treatment is also similar to findings in the rat [7,34,43,45,46], which are suggestive of neuronal atrophy that could be related to a decrease in phosphorylated neurofilament [45]. Finally, our morphological examination of DRGs and sciatic nerves revealed that many of the neurons were multinucleolated, that the nucleoli were eccentric and that rare nerve fibers presented mild signs of axonopathy. The morphometrical analysis showed the presence of somatic and nucleolar atrophy of DRG neurons. Altered DRG neuron morphology and morphometry have also been found in the rat following oxaliplatin treatment [44,47]. The underlying mechanisms of this phenomenon are unclear; although one study found that paclitaxel treatment induces nucleolar enlargement that can inhibit the neurotoxic effects of oxaliplatin [47].

Despite these decreases in NCV, the presence of mechanical and cold allodynia suggests an increase in peripheral nerve sensitivity [41,48]. The exact mechanism of oxaliplatin-induced hyperexcitability is unclear. One theory is that a metabolite of oxaliplatin, oxalate, may alter the functional properties of voltage-gated sodium channels, which are intrinsic to action potential generation, resulting in a prolonged open state of the channels and hyperexcitability of sensory neurons [49-51]. One possible mechanism underlying the disruption of voltage-gated sodium channel function is the calcium chelating effect of oxalate, which inhibits intracellular calcium-dependent mechanisms [13,50]. A second theory to explain oxaliplatin-induced hyperexcitability, with regard to cold allodynia, is that the sensitivity and expression levels of transient receptor potential melastatin 8 (TRPM8) and transient receptor potential ankyrin 1 (TRPA1) are increased after oxaliplatin treatment [15,52]. TRPM8 is only expressed in the DRG and responds to innocuous cool and noxious cold (<15C°) temperatures [53-55]. Anand et al. [52] found that oxaliplatin treatment increased the icilin response in cultured DRG neurons, suggesting that both the TRPM8 and TRPA1 channels are affected and Gauchan et al. [15] showed that blocking TRPM8 function by administering capsazepine inhibited the induction of cold allodynia after oxaliplatin treatment.

Given that the mice developed mechanical and cold allodynia after oxaliplatin treatment, our final area of inquiry was to examine the effects of oxaliplatin on the activity of wide dynamic range neurons in the spinal dorsal horn. A variety of neuropathic pain models exhibit increased evoked wide dynamic range (WDR) neuron activity after nerve injury [56-61]. In this study, mice that received chronic oxaliplatin treatment had a significant increase in wide dynamic range neuron activity compared to naive mice. Our findings are similar to those seen in other drug-induced neuropathy models [56,62] and may reflect physiological changes in the SDH as well as in primary afferents [58,63]. The oxaliplatin-induced increase in WDR neuron activity may be due to increased neurotransmitter release in the SDH [64-69], resulting in altered synaptic transmission [68,70] due to increased neurotransmitter levels. Increased levels of neurotransmitters, such as brain-derived neurotrophic factor (BDNF) can activate calcium permeable 2-amino-3-(5-methyl-3-oxo-1,2-oxazol-4-yl) propanoic acid (AMPA) channels, which increase excitatory synaptic activity and miniature excitatory post-synaptic currents (mEPSCs) in the SDH [68,70,71]. Further, increased neurotransmitter levels in the SDH can lead to a decreased frequency of inhibitory synaptic events, thus allowing for more excitatory activity [72]. The increase in WDR neuron activity is likely due to interactions between activated receptors and other signaling molecules [73-76], which results in longer lasting changes in SDH neurons such as what occurs in central sensitization [61].

It is well-documented that chemotherapeutic agents are toxic to the peripheral nervous system, though their mechanisms of action differ by class. However, many of the physiological mechanisms that underlie the development of chemotherapy-induced neurotoxicity remain unclear, regardless of the class of the drug. The platinum compounds, such as oxaliplatin, target the cell bodies of the neurons located in the DRG and disrupt the function of DNA [7,77]. In addition to the mechanisms discussed above, oxaliplatin may induce neurotoxicity by increasing p53 and p38 activity [78,79]. The taxanes [80,81], vinca alkaloids [82,83] and epothilones [84] all seem to exert their neurotoxic effects by inhibiting tubulin function and disrupting axonal transport and intracellular signaling processes. The symptoms associated with peripheral neurotoxicity vary by drug class but all have a significant deleterious effect on the quality of life of cancer patients that can become dose-limiting. Thus, it is important that research continues to increase the understanding of the mechanisms underlying the development of chemotherapy-induced neurotoxicity.

## Conclusions

The findings of this study provide evidence that our experimental paradigm produced a suitable mouse model of chronic oxaliplatin-induced neurotoxicity with mechanical and cold allodynia. Further, this study demonstrated that chronic oxaliplatin treat induces hyperexcitability of SDH neurons in addition to the neuropathological changes seen in the DRG and peripheral nerve. Therefore, this model can be used to further elucidate the mechanisms that underlie the development of chronic oxaliplatin-induced neuropathy.

## Methods

### 1. Experimental Design

The study was carried out using the same experimental protocol in 3 separate cohorts of animals (each comprised of a naïve group and a drug-treated group) for the NCV, spinal dorsal horn electrophysiology and behavioral experiments. The neuropathology and morphometry experiments were done using the same cohort of animals that underwent NCV testing. The animals were randomly divided into experimental groups. In each experiment, the drug-treated group was administered oxaliplatin 3.5 mg/Kg twice a week for a 4-week period with 3 or 4 days-elapsing between the administrations. The treatment schedule was chosen on the basis of literature data [40] and of our previous experiments (data not reported). Since the vehicle used has demonstrated to be non-toxic (data not reported), naïve control mice were left untreated.

### 2. Animals

Young adult female BALB/c mice (~20 g, Harlan, San Pietro al Natisone, Italy or Harlan Laboratory, Frederick, MD, USA) were used for the study. All mice were housed on a 12:12 h light:dark cycle with food and water available *ad libitum *in compliance with international policies (EEC, 1986; Italian, 1992; U.S. National Research Council, 1996). The International Association for the Study of Pain (IASP) guidelines for investigations of pain in animals was followed [85]. The Institutional Animal Care and Use Committee of the University of Maryland School of Medicine and the *ad hoc *committee for Animal Studies of the University of Milan-Bicocca approved these experiments. Throughout the duration of the study, the mice were visually examined daily for evidence of debilitation due to drug treatment, which is indicated by changes in their appearance (disheveled hair, weight loss and dehydration), behavior (decreased grooming, eating and drinking) and activity (decreased exploring and nesting). Any mouse that demonstrated signs of debilitation would be euthanized; however, no mice were prematurely euthanized in this study and all mice were euthanized at the completion of experiments.

### 3. Drug

Oxaliplatin (gifted by Debiopharm, Lausanne, Switzerland) solution was prepared immediately before each administration. Oxaliplatin was dissolved and diluted in 5% glucose solution. A 10 ml/kg volume, at the concentration of 3.5 mg/kg, was administered intravenously by tail vein injection. Using Du Bois's formula to calculate human body surface area, this dose is equivalent to 130 mg/m^2 ^per administration and a cumulative dose of 1080 mg/m^2 ^after 8 cycles. This dose was chosen based on previous pilot studies (data not reported).

### 4. Anesthesia

For the NCV measurement and electrophysiological recording, anesthesia was induced in a chamber with 3% isoflurane carried in medical air followed by 1-1.5% isoflurane delivered by nose cone for maintenance throughout the procedures, which was adequate to suppress the corneal blink response and any withdrawal response to a noxious stimulus. Additionally, prior to the laminectomy surgery for the spinal cord electrophysiological recordings, mice were given an intraperitoneal injection of Pentobarbital 40 mg/kg and a local subcutaneous injection of marcaine 0.5% (75-100 μl) at the surgical site. Throughout both procedures, the mice continued to breathe spontaneously at ~2-3 Hz and the heart rate was maintained at ~9-10 Hz (MouseOx, STARR Life Sciences Corp., Oakmont, PA). The core body temperature was continuously monitored and maintained at ~37°C by a heating pad (Homeothermic Blanket System, Harvard Apparatus, Holliston, MA) to avoid isoflurane-induced hypothermia.

### 5. Tail Vein Injection

The mice were placed in a Broome Style Rodent Restrainer (Plas Labs, Lansing, MI) with the tail extending from the end. The tail was vasodilated by immersion in a warm water bath (40-42°C) for 15-30 seconds prior to injection. A 100 μl Hamilton syringe with a 1/2" 30 g needle was used for the injection. The lateral tail vein was located and the tail was firmly immobilized between the thumb and forefinger. The needle was inserted, bevel up, at a 10° angle in the rostral direction. The solution was injected slowly while watching closely for the vein to blanch, with no detectable swelling of the tail near the injection site. When the needle was removed, pressure was applied to the injection site for 15-30 seconds to stop bleeding and minimize hematoma formation.

### 6. Assessment of Oxaliplatin-Induced Peripheral Neurotoxicity

#### 6.1. NCV Measurement

The investigator was blind to the animal conditions for these experiments. Four days after the last dose of oxaliplatin, caudal and digital NCVs were measured to determine peripheral sensory/motor nerve functional status. Using electromyography (Myto2 ABN Neuro, Firenze, Italy), caudal NCV was determined by placing two proximal recording needle electrodes (one anode and one cathode) on the tail and two stimulating needle electrodes 3.5 cm distal to the recording electrodes. Similarly, the digital NCV was determined in the hind paw by placing the recording electrodes near the ankle and the stimulating electrodes in the fourth toe near the digital nerve. The optimal intensity, frequency and duration of stimulation (10mA, 0.75 sec and 0.04 msec, respectively) were determined prior to this study to generate high-quality results. Both the caudal and digital NCVs were calculated as a ratio (m/sec) of the distance (cm) between stimulating and recording electrodes and the time latency (sec.) from the stimulus artifact to the onset of the elicited action potential. Serial stimulations with different amplitudes (2-20 mA) were performed to achieve the maximal action potential feedback. Ten responses per stimulation amplitude were averaged for each recording.

#### 6.2. Neuropathological Analysis of DRG and Sciatic Nerve

The investigator was blind to the nerve and DRG conditions for these analyses. Three animals from each group were sacrificed four days after the last oxaliplatin dose. The left sciatic nerves and the L4-L5 DRGs were harvested and processed following previously reported protocols, resin embedded and sectioned for light and electron microscope analysis [7,16,17,86]. For the light microscopy analysis, 1 μm semithin sections were stained with toluidine blue and examined using a Nikon Eclipse E200 light microscope (Nikon, Firenze, Italy). For the electron microscopy analysis, ultrathin sections (80 nm) counterstained with uranyl acetate and lead citrate, were examined with a Philips CM 10 transmission electron microscope (Philips, Eindhoven, Netherlands).

#### 6.3. Morphometric Analysis of DRG Neurons

The investigator was blind to the experimental conditions of the tissue for these experiments. One micron thick semithin sections stained with toluidine blue were used for morphometric examination of the DRGs from control (n = 3) and oxaliplatin-treated mice (n = 3). Only the cells where the nucleolus was included in the section plane of 50 μm-spaced out sections were considered to avoid the overlapping of the same cell bodies and they were analyzed with computer-assisted image analysis (ImageJ, NIH) to measure the soma, nucleus and nucleolus size of at least 300 DRG neurons per mouse following previously reported methods used in [16].

### 7. Assessment of Oxaliplatin-Induced neuropathic Pain

#### 7.1. Behavioral Testing - Mechanical Allodynia

The investigator was blind to the animal conditions for these experiments. The nocifensive behavior of paw withdrawal from a mechanical stimulus was used to assess the development of mechanical allodynia. The mice were placed in individual Plexiglas cubicles on a wire mesh platform, and allowed to acclimate for approximately one hour, during which exploratory and grooming activity ended. A series of von Frey filaments (Touch Test Sensory Evaluator Kit, myNeurolab.com, St. Louis, MO), with bending forces that ranged from 0.04 g to 1.40 g, was used to deliver the mechanical stimuli. Each filament was applied to the hind paw until the filament just bent and was held in place for 5 seconds or until the mouse withdrew his paw. Each filament was tested 5 times on each hind paw. A positive response to the stimulus was defined as a brisk withdrawal, with or without shaking or licking, of the hind paw during or immediately upon removal of the filament application. The mechanical stimuli were applied to the plantar surface of each hind paw, starting with the 0.4 g filament [87]. If the 0.4 g filament elicited 3 positive responses out of 5 trials, then the mouse was tested moving downward through the series to the 0.04 g filament and the number of withdrawals was recorded for each filament. If the 0.4 g filament did not elicit 3 positive responses, then the mouse was tested moving upward through the series to the 1.4 g filament and the number of withdrawals was recorded for each filament. Threshold was defined as the filament with the lowest bending force that elicited at least 3 positive responses out of 5 trials.

#### 7.2. Behavioral Testing - Heat Hyperalgesia

The investigator was blind to the animal conditions for these experiments. Using an incremental hot plate (PE34, IITC Life Sciences; starting temperature 30°C; ramp set at the maximum rate of 10°C/min), the nocifensive behavior of licking the hind paw was used to identify the threshold for noxious heat. Each mouse was placed in a Plexiglas cylinder on the metal plate of the instrument and allowed to acclimate for 30-60 seconds prior to activating the instrument. The moment the mouse licked a hind paw, the stimulus was terminated, the plate temperature immediately returned to 30°C and the mouse was returned to its home cage. The temperature of the plate at the time when the licking occurred was recorded as the outcome measure. This was repeated after at least 30 minutes elapsing between the two trials and the mean of the two temperatures was determined.

#### 7.3. Behavioral Testing - Cold Allodynia

The investigator was blind to the animal conditions for these experiments. The experimental procedures for the cold allodynia testing were the same as those used for the heat hyperalgesia testing. Using an incremental cold plate (PE34, IITC Life Sciences; starting temperature 30°C; ramp set at the maximum rate of 10°C/min), the nocifensive behavior of jumping was used to identify the threshold for noxious cold. The temperature of the plate at the time when the mouse jumped was recorded as the outcome measure. This was repeated after at least 30 minutes elapsing between the two trials and the mean of the two temperatures was determined.

### 8. Electrophysiology

The investigator was blind to the animal conditions for these experiments. Extracellular electrophysiological recording was done to measure the activity of wide dynamic range neurons in the spinal dorsal horn. A laminectomy was performed at the level of the T13-L2 vertebrae to expose the L4-L5 spinal segment 2 days after the final dose of oxaliplatin. The vertebrae immediately adjacent to the laminectomy site were clamped firmly to immobilize the spinal column and minimize movement of the spinal cord during recording. A pool around the laminectomy site was formed with 5% agar, the dura was carefully removed from the cord and the pool was filled with warm 0.9% saline. Extracellular potentials were recorded using a fine-tip (<1.0 μm) tungsten microelectrode (10 MΩ, Frederick Haer Co., Brunswick, ME), then amplified and filtered using standard electrophysiological techniques. Under magnification with a surgical dissecting microscope, the electrode tip was placed on the dorsal surface of the spinal cord in a vertical position and advanced 400-650 μm electronically (Model 660 micropositioner, David Kopf Instruments, Tujunga, CA). The Cambridge Electronics Design (Cambridge, UK) micro 1401 and Spike2 (v6.0) software were used to acquire and digitize activity. The search stimulus to identify a unit was continuous stroking with a sable-hair brush on the plantar surface of the hind paw ipsilateral to electrode placement. To optimize the amplitude of an identified neuron, the electrode was moved in the dorsal-ventral plane. When a unit was isolated and its amplitude optimized, the response to mechanical stimuli was recorded. First, the response to 10-seconds of continuous brushing with the sable-hair brush was recorded. This was followed by recording the responses to 2 seconds of blunt pressure applied with a wooden probe (10 mm diameter), a 2-second pinch with blunt forceps and then a drop of acetone applied to the plantar surface of the hind paw with a 50 μl Hamilton syringe. At least 30 seconds elapsed between stimulus applications. Two recordings along the rostral-caudal axis (moving in the rostral to caudal direction) were made from most mice. Neuronal activity was discriminated, sorted and analyzed by principal components analysis offline using SciWorks (v7.0, Datawave Technologies, Berthoud, CO) to generate a peristimulus histogram with 1-second bins. Stimulus-evoked activity was quantified by calculating the number of spikes/1-second bin during the stimulation.

### 9. Data Analysis

The body weight changes, NCV, morphometric and electrophysiology data are presented as the mean ± S.E.M. The body weight changes were analyzed by ANOVA with repeated measures. The NCV, morphometry and electrophysiology data were analyzed for significant differences between 2 groups by Student's t-test. The thermal (hot & cold) behavioral data are presented as the mean ± S.E.M. The data were analyzed for significant differences between 2 groups by Student's t-test and within groups by ANOVA with repeated measures. The mechanical behavioral data are presented as ordinal data and were analyzed using the Mann-Whitney U-test to identify differences between 2 groups and the Friedman Test to identify differences with repeated measures within groups [79]. In all cases, a priori p < 0.05 was considered significant.

## List of Abbreviations

NCV: Nerve Conduction Velocity; DRG: Dorsal Root Ganglia; SDH: Spinal Dorsal Horn; OHP: Oxaliplatin; OIPN: Oxaliplatin-Induced Peripheral Neuropathy; WDR: Wide Dynamic Range.

## Competing interests

The authors declare that they have no competing interests; however, G.C. has served as a consultant to Delbiopharm SA, the original manufacturer of oxaliplatin.

## Authors' contributions

CLR performed the behavioral data collection, administered the drug injections, analyzed and interpreted the behavioral and electrophysiological data, contributed intellectually to the conceptualization and design of the project and drafted the manuscript in conjunction with VAC. VAC carried out the experimental procedures concerning the pharmacological treatments, assessed the general toxicity of the drug, perform the neurophysiological studies and the morphological and morphometrical analysis. VAC also drafted the manuscript with CLR. PR conducted the electrophysiological recordings of SDH, assisted with drug injections. DG assisted with behavioral testing, data collection and monitoring the general condition of the mice. SGD contributed intellectually to the conceptualization and design of the project and critically revising the draft of the manuscript.GC contributed intellectually to the conceptualization and design of the project and critically revising the draft of the manuscript. All authors read and approved the manuscript.
